# Long COVID and psychosocial factors among middle-aged and older adults. Results of the nationally representative German Ageing Survey

**DOI:** 10.1007/s40520-025-03246-7

**Published:** 2025-11-05

**Authors:** André Hajek, Christine Blome, Dong Keon Yon, Pinar Soysal, Razak M. Gyasi, Karl Peltzer, Supa Pengpid, Hans-Helmut König

**Affiliations:** 1 Department of Health Economics and Health Services Research, University Medical Center Hamburg-Eppendorf, Hamburg Center for Health Economics, Hamburg, Germany; 2 Institute for Health Services Research in Dermatology and Nursing (IVDP), University Medical Center Hamburg-Eppendorf, Hamburg, Germany; 3 Center for Digital Health, Medical Science Research Institute, Kyung Hee University College of Medicine, Seoul, South Korea; 4 Department of Geriatric Medicine, Faculty of Medicine, Bezmialem Vakif University, Istanbul, Turkey; 5 African Population and Health Research Center, Nairobi, Kenya; 6 National Centre for Naturopathic Medicine, Faculty of Health, Southern Cross University, Lismore, NSW Australia; 7 Department of Health Education and Behavioral Sciences, Faculty of Public Health, Mahidol University, Bangkok, Thailand; 8 Department of Psychology, University of the Free State, Bloemfontein, South Africa; 9 Department of Medical Research, China Medical University Hospital, China Medical University, Taichung, Taiwan; 10 Department of Public Health, Sefako Makgatho Health Sciences University, Pretoria, South Africa; 11 Department of Healthcare Administration, College of Medical and Health Science, Asia University, Taichung, Taiwan

**Keywords:** Loneliness, Perceived social isolation, Social exclusion, Depressive symptoms, Mental health, Life satisfaction, Long COVID, Post-COVID syndrome, Post-Acute COVID-19 syndrome

## Abstract

**Background:**

In addition to the physical symptoms, long COVID can cause considerable psychological burden.

**Aims:**

To investigate the association of long COVID with depressive symptoms, loneliness, perceived social isolation and life satisfaction (also stratified by sex).

**Methods:**

Data from the most recent eighth wave of the nationally representative German Ageing Survey was used, encompassing community-dwelling individuals 43 years to 90 years, *n* = 4,017 individuals in the analytic sample). Psychometrically sound tools were used to quantify the outcomes. Physician-diagnosed long COVID was used as independent variable. Adjusted (weighted) linear regressions with cluster-robust standard errors were used. Robustness checks were conducted.

**Results:**

Regressions adjusted for sociodemographic and lifestyle-related covariates showed that individuals with long COVID had consistently worse psychosocial outcomes compared to individuals without long COVID. However, after additionally adjusting for health-related covariates, only the association between long COVID and perceived social isolation remained significant (β = 0.29, *p* < 0.001). Stratified by sex, long COVID was significantly associated with higher social isolation scores among women (β = 0.37, *p* < 0.001), but not among men in the fully adjusted models.

**Discussion:**

Even after adjusting for a wide array of covariates, findings suggest that (female) individuals with long COVID have stronger feelings of not belonging to the society (compared to individuals without long COVID).

**Conclusions:**

It may be beneficial to find ways to help such individuals feel included in society.

**Supplementary Information:**

The online version contains supplementary material available at 10.1007/s40520-025-03246-7.

## Introduction

The COVID-19 pandemic threw the world into turmoil at the beginning of the 2020s. Even in the mid-2020s, societies and scientists are still dealing with and investigating the consequences of the pandemic. In particular, long COVID (also: Post-COVID Syndrome, Post-COVID-19 condition, Post-acute sequelae of COVID-19, or Post-Acute COVID-19 Syndrome) should be mentioned here. According to Nalbandian et al. [1], long COVID is a multisystemic condition “characterized by persistent symptoms and/or delayed or long-term complications beyond 4 weeks from the onset of symptoms” (p. 601).

Long COVID is characterized by a plethora of physical symptoms across multiple organ systems [2] (see also: [3]). A previous review identified cognitive and neurological symptoms such as dizziness, memory loss, cognitive impairment, and fatigue as key features of the condition [2]. A very recent previous systematic review and meta-analysis revealed that one in four people with long COVID experience depression – which is also common two years after infection [4]. In addition to physical and mental health symptoms, long COVID is also associated with psychosocial factors. For example, individuals with long COVID often report stigmatization [5, 6]. A previous descriptive study [7] using data from a convenience sample of individuals who reported experiencing long COVID (but without a control group of individuals without COVID) also showed that 19% of the participants reported being lonely often or always (see also: [8]). Moreover, 39% of the participants reported feeling isolated often and 32% of the participants felt left out often [7]. Another study showed that life satisfaction (single item) was significantly negatively associated with a count of long COVID symptoms based on a small sample from a single clinic [9].

Previous studies of long COVID and psychosocial outcomes are limited by the fact that they mainly rely on small convenience samples, do not include a control group of people without long COVID and do not comprehensively encompass psychosocial outcomes using psychometrically sound tools. More specifically, very little is known about the association of long COVID with life satisfaction, loneliness and perceived social isolation. To address these gaps in knowledge, our aim was to investigate the association of long COVID with several psychosocial outcomes (in terms of depressive symptoms, loneliness, perceived social isolation and life satisfaction). We used data from a large, nationally representative sample of community-dwelling individuals aged 43 years and over, using psychometrically sound tools to quantify the outcomes of interest. A control group of individuals without long COVID was also included. Sex-stratified analyses were additionally conducted because of possible sex-related differences in coping strategies and experiences within society when having long COVID [10–12]. For example, a previous qualitative study (based on 15 adult women living in the United States) showed that long COVID impacted their social life by increasing, among other things, social stigma, physical limitations and family-work conflicts [10]. Women in particular may face stigma. For example, they may not be taken seriously by healthcare professionals when they report symptoms, due to gender biases [13].

Our findings are important to better understand long COVID in the context of psychosocial variables. This can be relevant for various professional groups such as physicians, healthcare professionals and public health experts.

In order to better contextualize our present findings, we briefly outline the COVID-19 situation in Germany at the time of data collection (late 2022 to mid-2023): During this period, Germany transitioned into an endemic phase of the virus. Although Omicron subvariants dominated, the severity of individual cases decreased overall due to high population immunity. Thus, several restrictions such as testing requirements or mask mandates on public transport were steadily eased. Moreover, the World Health Organization officially lifted coronavirus health emergency in May 2023.

## Methods

### Sample

For this current study, data were taken from Wave 8 (due to reasons of data availability) of the German Ageing Survey (DEAS), conducted between the end of 2022 and mid-2023. In general, the DEAS study is nationally representative for community-dwelling individuals aged 40 years and older in Germany. Starting with wave 3, people were surveyed every 3 years. Wave 8 was a pure panel wave, which means that individuals were only included when they already participated before. Therefore, individuals were at least 43 years old in wave 8.

The DEAS study covers numerous topics such as labor force participation, retirement transitions, health and well-being. Participants were surveyed through both telephone (Computer-Assisted Telephone Interviewing, CATI) and face-to-face (Computer-Assisted Personal Interviewing, CAPI) methods. The overall response rate was approximately 62%, with each interview lasting around 81 min on average. Following the interview, participants had the option to complete an additional questionnaire (either by mail or online), predominantly covering more sensitive topics such as life satisfaction or loneliness.

### Outcome: psychosocial outcomes

Depressive symptoms were quantified using the Center for Epidemiologic Studies Depression Scale (CES-D) [14]. The CES-D consists of 15 items (each ranging from 0 to 3). Therefore, the sum score ranges from 0 to 45 (with higher values reflecting more depressive symptoms). In our study, Cronbach’s alpha was 0.86 (McDonald’s omega was 0.87).

The widely recognized De Jong Gierveld tool [15] was employed to assess loneliness in this study, consisting of six items. An overall score was derived by averaging all six items. It ranges from 1 to 4, with higher values indicating more loneliness. In our study, Cronbach’s alpha was 0.84 (McDonald’s omega was also 0.84).

To quantify perceived social isolation, a four-item tool developed by Bude and Lantermann [16] was used. The total score was derived by averaging responses across all four items. The final score varies from 1 to 4 (higher scores indicate higher levels of perceived social isolation). Both, Cronbach’s alpha and McDonald’s omega equaled 0.87.

The established Satisfaction with Life Scale (SWLS) [17] was used to quantify life satisfaction. The SWLS consists of five items. The resulting sum score ranges from 1 to 5 (whereby higher values indicate higher levels of life satisfaction). In our study, Cronbach’s alpha and McDonald’s omega both equaled 0.85.

### Independent variables of interest: long COVID

From a predefined list of various health conditions, participants were asked to indicate those illnesses with which they had received a formal diagnosis from a physician. The occurrence of long COVID was identified based on respondents’ answers to this specific long COVID question. Individuals were also asked about: increased blood fat levels, cholesterol levels; diabetes, increased blood sugar levels; high blood pressure; heart attack, angina pectoris; cardiac insufficiency including coronary artery disease; stroke; circulatory disorders in the brain; circulatory disorders in the legs; joint degeneration (arthrosis of the hips, knee joints or of the spine); osteoporosis; inflammatory joint or spinal disease (arthritis or rheumatoid arthritis); chronic lung disease (e.g., chronic bronchitis, pulmonary emphysema); cancer, malignant tumor (including leukemia); gastric ulcer, duodenal ulcer; incontinence; mental illness (e.g. panic attacks, depression, psychosis); Parkinson’s disease; glaucoma or macular degeneration. The selection of illnesses was guided by established frameworks, including the Charlson comorbidity index [18]. Additional consultations with geriatric specialists were conducted to ensure a comprehensive and clinically relevant selection of diseases.

### Covariates

As sociodemographic covariates, age, sex (men, women), marital status (five categories: married and living together with spouse, married but living separated from spouse, single, widowed, divorced), labor force participation (three categories: employed, retired, other), and education (International Standard Classification of Education [ISCED]-classification [19]; three categories: low, medium or high) were included in the regression analysis.

As lifestyle-related covariates, alcohol consumption, sports activity (six categories for both variables: daily; several times a week; once a week; 1–3 times a month; less often; never), and smoking status (four categories: yes, daily; yes, sometimes; no, not anymore, no, never) were used in the regression analysis.

As health-related covariates, self-rated health, physical functioning, and the number of chronic conditions were included in the regression analysis. Self-rated health was quantified using a single-item tool ranging from 1 = very good to 5 = very poor. Physical functioning was quantified using the respective SF-36 subscale (10 items) [20], ranging from 0 to 100 with higher values corresponding to better physical functioning. The count score of eleven chronic conditions (cardiac and circulatory disorders; bad circulation; joint, bone, spinal and back problems; respiratory problems, asthma, shortness of breath; stomach and intestinal problems; cancer; diabetes; gall bladder, liver or kidney problems; bladder problems; eye problems, vision impairment; ear problems, hearing problems) reflects the presence or absence of such conditions.

### Statistical analysis

Sample characteristics are shown first. Subsequently, unadjusted and adjusted linear regressions were estimated, also stratified by sex. Sampling weights were applied and cluster-robust standard errors were calculated, clustering standard errors at the level of the primary sampling unit (to account for the complex survey design and non-response). The initial adjusted model accounted for a range of sociodemographic covariates. This was followed by the inclusion of lifestyle-related factors into the model. Finally, health-related covariates were added.

Given that the proportion of missing data was marginal (markedly below 1% in most variables, and overall, only 3.2% of observations have at least one missing value), listwise deletion was employed for the main analysis, which included an analytic sample of 4,017 individuals (fully-adjusted model with depressive symptoms as outcome). However, to assess the robustness of our results, we also conducted a regression analysis with full-information maximum likelihood (FIML) to address missing values [21].

McDonald’s omega was computed based on a tool created by Shaw [22]. Statistical significance was established at a p-value below 0.05. All analyses were performed using StataNow 19.5 MP-Parallel Edition (StataCorp, College Station, Texas).

## Results

### Sample characteristics

Table 1 shows the analytic sample (*n* = 4,017 individuals), also stratified by long COVID. Among the total sample, the mean age was 69.1 years (standard deviation (SD): 11.1 years, 43 to 90 years; 52.2% were female). The average depressive symptoms score was 6.4 (SD: 6.0), the average loneliness score was 1.7 (SD: 0.5), the average perceived social isolation score was 1.6 (SD: 0.6), and the average life satisfaction score was 3.9 (SD: 0.7). Individuals with and without long COVID substantially differed in terms of health-related factors in particular. Further details are displayed in Table 1.

**Table 1:** Sample characteristics, overall and by sex (analytic sample with *n* = 4,017 individuals)

Variables	Absence of long COVID	Presence of long COVID	Total	*P*-value
N (%)	3,890 (96.8)	127 (3.2)	4,017 (100.0)	
Age: Mean (SD)	69.1 (11.1)	68.6 (11.8)	69.1 (11.1)	0.62
Sex: N (%)				0.22
Men	1,868 (48.0)	54 (42.5)	1,922 (47.8)	
Women	2,022 (52.0)	73 (57.5)	2,095 (52.2)	
Marital status: N (%)				0.28
Married, living together with spouse	2,603 (66.9)	91 (71.7)	2,694 (67.1)	
Married, living separated from spouse	36 (0.9)	3 (2.4)	39 (1.0)	
Divorced	386 (9.9)	12 (9.4)	398 (9.9)	
Widowed	578 (14.9)	13 (10.2)	591 (14.7)	
Single	287 (7.4)	8 (6.3)	295 (7.3)	
Employment status: N (%)				0.15
Employed	1,098 (28.2)	39 (30.7)	1,137 (28.3)	
Retired	2,615 (67.2)	78 (61.4)	2,693 (67.0)	
Other: not employed	177 (4.6)	10 (7.9)	187 (4.7)	
Education: N (%)				0.69
Low	132 (3.4)	3 (2.4)	135 (3.4)	
Medium	1,796 (46.2)	56 (44.1)	1,852 (46.1)	
High	1,962 (50.4)	68 (53.5)	2,030 (50.5)	
Smoking behavior: N (%)				0.10
Yes, daily	411 (10.6)	5 (3.9)	416 (10.4)	
Yes, sometimes	152 (3.9)	5 (3.9)	157 (3.9)	
No, not anymore	1,505 (38.7)	56 (44.1)	1,561 (38.9)	
No, never	1,822 (46.8)	61 (48.0)	1,883 (46.9)	
Frequency of sports activity: N (%)				0.09
Daily	404 (10.4)	6 (4.7)	410 (10.2)	
Several times a week	1,214 (31.2)	52 (40.9)	1,266 (31.5)	
Once a week	675 (17.4)	17 (13.4)	692 (17.2)	
1–3 times a month	225 (5.8)	7 (5.5)	232 (5.8)	
Less often	391 (10.1)	10 (7.9)	401 (10.0)	
Never	981 (25.2)	35 (27.6)	1,016 (25.3)	
Alcohol intake: N (%)				0.38
Daily	422 (10.8)	9 (7.1)	431 (10.7)	
Several times a week	982 (25.2)	32 (25.2)	1,014 (25.2)	
Once a week	645 (16.6)	27 (21.3)	672 (16.7)	
1–3 times a month	532 (13.7)	12 (9.4)	544 (13.5)	
Less often	899 (23.1)	33 (26.0)	932 (23.2)	
Never	410 (10.5)	14 (11.0)	424 (10.6)	
Number of chronic conditions: Mean (SD)	2.7 (2.0)	3.5 (2.2)	2.8 (2.0)	< 0.0.001
Self-rated health: Mean (SD)	2.5 (0.8)	2.9 (0.8)	2.5 (0.8)	< 0.0.001
Physical functioning: Mean (SD)	81.4 (22.6)	72.5 (27.5)	81.1 (22.8)	< 0.0.001
Depressive symptoms: Mean (SD)	6.4 (6.0)	8.9 (6.8)	6.4 (6.0)	< 0.0.001
Loneliness: Mean (SD)	1.7 (0.5)	1.8 (0.5)	1.7 (0.5)	0.24
Perceived social isolation: Mean (SD)	1.6 (0.6)	1.7 (0.6)	1.6 (0.6)	< 0.01
Life satisfaction: Mean (SD)	3.9 (0.7)	3.7 (0.7)	3.9 (0.7)	0.02

*P*-values are based on independent t-tests of Chi²-tests, as appropriate. Weights were not used. Number of chronic conditions ranges from 0 to 11, self-rated health ranges from 1 (very good) to 5 (very poor), physical functioning ranges from 0 to 100 (with higher scores reflecting better physical functioning), depressive symptoms range from 0 to 45 (with higher values reflecting more depressive symptoms), loneliness ranges from 1 to 4 (with higher values reflecting higher loneliness levels), perceived social isolation ranges from 1 to 4 (with higher values reflecting higher perceived social isolation levels), and life satisfaction ranges from 1 to 5 (with higher values reflecting higher life satisfaction levels)

### Regression analysis

Results of unadjusted and adjusted linear regressions for the association of long COVID with depressive symptoms, loneliness, perceived social isolation, and life satisfaction among the total sample are shown in Tables 2, 3, 4 and 5 respectively.

**Table 2:** Association of long COVID with depressive symptoms among the total sample. Results of linear regressions (DEAS, wave 8, weighted)

	Depressive symptoms	Depressive symptoms	Depressive symptoms	Depressive symptoms
Long COVID: Presence (reference category: absence)	4.12*	3.88*	3.95*	1.66
	(0.73–7.50)	(0.86–6.90)	(0.92–6.98)	(−0.92–4.23)
Sociodemographic covariates		✓	✓	✓
Lifestyle-related covariates			✓	✓
Health-related covariates				✓
Individuals	4,147	4,134	4,094	4,017
R²	0.01	0.07	0.10	0.29

Comments: Unstandardized beta coefficients are shown, with 95% CI in parentheses; *** *p* < 0.001, ** *p* < 0.01, * *p* < 0.05, + *p* < 0.10

Sociodemographic covariates include: age, sex, marital status, labor force participation, and education; lifestyle-related covariates include: alcohol intake, sports activity, and smoking behavior; health-related covariates include: self-rated health, physical functioning, and the number of chronic conditions

**Table 3:** Association of long COVID with loneliness among the total sample. Results of linear regressions (DEAS, wave 8, weighted)

	Loneliness	Loneliness	Loneliness	Loneliness
Long COVID: Presence (Reference category: Absence)	0.21*	0.22**	0.24**	0.13
	(0.04–0.37)	(0.06–0.37)	(0.08–0.41)	(−0.04–0.30)
Sociodemographic covariates		✓	✓	✓
Lifestyle-related covariates			✓	✓
Health-related covariates				✓
Individuals	4,114	4,101	4,065	3,989
R²	0.00	0.03	0.07	0.13

Comments: Unstandardized beta coefficients are shown, with 95% CI in parentheses; *** *p* < 0.001, ** *p* < 0.01, * *p* < 0.05, + *p* < 0.10

Sociodemographic covariates include: age, sex, marital status, labor force participation, and education; lifestyle-related covariates include: alcohol intake, sports activity, and smoking behavior; health-related covariates include: self-rated health, physical functioning, and the number of chronic conditions

**Table 4:** Association of long COVID with perceived social isolation among the total sample. Results of linear regressions (DEAS, wave 8, weighted)

	Perceived social isolation	Perceived social isolation	Perceived social isolation	Perceived social isolation
Long COVID: Presence (Reference category: Absence)	0.39***	0.40***	0.41***	0.29***
	(0.24–0.53)	(0.26–0.54)	(0.26–0.57)	(0.13–0.45)
Sociodemographic covariates		✓	✓	✓
Lifestyle-related covariates			✓	✓
Health-related covariates				✓
Individuals	4,121	4,108	4,068	3,993
R²	0.01	0.06	0.08	0.15

Comments: Unstandardized beta coefficients are shown, with 95% CI in parentheses; *** *p* < 0.001, ** *p* < 0.01, * *p* < 0.05, + *p* < 0.10

Sociodemographic covariates include: age, sex, marital status, labor force participation, and education; lifestyle-related covariates include: alcohol intake, sports activity, and smoking behavior; health-related covariates include: self-rated health, physical functioning, and the number of chronic conditions

**Table 5:** Association of long COVID with life satisfaction among the total sample. Results of linear regressions (DEAS, wave 8, weighted)

	Life satisfaction	Life satisfaction	Life satisfaction	Life satisfaction
Long COVID: Presence (Reference category: Absence)	−0.24*	−0.26*	−0.29**	−0.08
	(−0.45 - −0.03)	(−0.47 - −0.04)	(−0.51 - −0.08)	(−0.33–0.17)
Sociodemographic covariates		✓	✓	✓
Lifestyle-related covariates			✓	✓
Health-related covariates				✓
Individuals	4,128	4,115	4,074	3,999
R²	0.00	0.11	0.17	0.32

Comments: Unstandardized beta coefficients are shown, with 95% CI in parentheses; *** *p* < 0.001, ** *p* < 0.01, * *p* < 0.05, + *p* < 0.10

Sociodemographic covariates include: age, sex, marital status, labor force participation, and education; lifestyle-related covariates include: alcohol intake, sports activity, and smoking behavior; health-related covariates include: self-rated health, physical functioning, and the number of chronic conditions

Unadjusted regression analyses revealed that individuals with long COVID had consistently worse psychosocial outcomes (in terms of more depressive symptoms, higher loneliness levels, higher perceived social isolation levels, and lower life satisfaction levels) compared to individuals without long COVID. These associations remained significant even when adjusted for sociodemographic and lifestyle-related factors. However, after additionally adjusting for health-related covariates, only the association between long COVID and perceived social isolation remained significant (β = 0.29, *p* < 0.001).

In the fully adjusted and sex-stratified models (see Supplementary Table 1), long COVID was significantly associated with higher social isolation scores among women (β = 0.37, *p* < 0.001), but not men. The corresponding interaction term (sex x long COVID) was β = 0.27 (*p* = 0.17). In a sensitivity analysis with FIML (rather than listwise deletion), the findings remained virtually the same in terms of significance and effect size.

## Discussion

Based on data from a nationally representative sample of community-dwelling individuals aged 43 years and older in Germany, the aim of this study was to investigate the association of long COVID with psychosocial outcomes. Our key findings were: After adjustment for sociodemographic and lifestyle factors, individuals with long COVID consistently showed poorer psychosocial outcomes (compared to individuals without long COVID). However, after further controlling for health-related covariates, only the association with perceived social isolation remained significant. When stratified by sex, this association was significant among women but not among men in the fully adjusted regression models. Several previous studies have focused on the association of long COVID with mental health in particular (and were often restricted by small, non-generalizable samples and missing healthy control groups). Our study considerably extends the present knowledge by looking comprehensively at psychosocial outcomes (using valid tools to quantify life satisfaction, loneliness, and perceived social isolation) based on data from a large, generalizable sample.

In our study, long COVID was significantly associated with depressive symptoms only when it was not adjusted for health-related covariates. To put it in perspective: At first glance, mental health seems to be well-researched in long COVID [23, 24]. However, a closer look reveals that there are often methodological limitations (e.g. a control group of people without long COVID is often missing) [25]. In this respect, many results of earlier studies must be interpreted with caution. For example, a former systematic review and meta-analysis [26] identified a pooled prevalence of probable depression of about 23% (95% CI: 20–26%) among patients with long COVID. However, it is worth noting that such prevalence is comparable to prevalence rates of general adult population samples at different times during the pandemic, quantified with comparable tools [27, 28]. Studies explicitly examining the association of long COVID and mental health yielded mixed results, often depending on the covariates included (see: [29, 30]). It is worth noting that it was adjusted for other health-related factors (such as self-rated health) in our current study. This may also capture symptoms that can result from depression or long COVID, such as fatigue or trouble concentrating. This may explain why long COVID was not significantly associated with depressive symptoms in our fully-adjusted model. More generally and as expected, adjustment of health-related covariates unsurprisingly markedly attenuated the associations of interest. Given the physical and mental impairments often found in people with long COVID, it is very plausible that such adjustment attenuated the associations of interest.

In this study, long COVID was not significantly associated with life satisfaction in the fully-adjusted model. One former study has shown that life satisfaction (based on a single-item) was associated with the number of long COVID symptoms (*p* = 0.04) after adjusting for age, number of symptoms during acute infection, and depression based on 166 patients of a clinic of Semmelweis University (Hungary) [9]. Another study showed that self-reported long COVID was significantly associated with lower life satisfaction (also using the SWLS) based on data from community-dwelling individuals aged 20 to 50 years contracting COVID-19 before vaccination in Galveston county (southeast Texas, United States) [31]. Differences between those two studies and our current findings may be mainly explained by differences in the model specification (i.e., different covariates), composition of the sample, as well as in the measurement of long COVID and life satisfaction. Even though long COVID can be a major disruption in life (e.g. for working life and everyday activities), it does not seem to contribute to the cognitive evaluation of life as a whole the way other critical life events do [32].

Our study showed that while long COVID was not significantly associated with loneliness, it was significantly associated with perceived social isolation in the fully-adjusted model. Since there is an almost lack of studies examining such associations, it is difficult our present findings with previous studies. Only one previous study showed an association between having long COVID (compared to never having long COVID) and moderate-to-high loneliness [8]. We assume that many individuals with long COVID are still connected to their existing social network such as close friends and family members. However, such individuals may feel excluded from wider society, for example because their symptoms of illness are ridiculed, psychologized, or not taken recognized or taken seriously [33–35]. Many report encountering misunderstanding and comments suggesting that they should not make such a fuss about their long COVID symptoms [33, 34]. Individuals with long COVID may also feel stigmatized from society. The high perceived social isolation levels experienced by women with long COVID may be partly due to societal gender roles, which can result in their symptoms being dismissed, not taken seriously or misinterpreted as laziness [10] even more often than in men. However, these are speculative explanations that warrant further examination in future research.

When interpreting the current results, it is important to consider both their strengths and limitations. Data came from a large, nationally representative sample of individuals aged ≥ 43 years living in private households across Germany. In general, psychometrically sound tools were used to quantify the outcomes. A wide array of covariates was incorporated in the regression analysis. Additionally, sampling weights were applied to address the survey design and non-response. FIML was used in a robustness check to address missing values. Physician-diagnosed long COVID was reported. However, the association of the severity of long COVID with psychosocial outcomes should be further explored in future studies. The cross-sectional design of our study does not allow any conclusions to be drawn about the long-term psychosocial consequences.

Even after adjustment was made for a wide array of covariates, findings suggest that (female) individuals with long COVID (in comparison to individuals without long COVID) have stronger feelings of not belonging to society. Further research is needed on the exact reasons for this experience. It may be beneficial to find ways to help such individuals feel included in society. For example, efforts to improve the resilience of female individuals with long COVID may be beneficial. More precisely, self-help groups and peer support can provide a valuable opportunity to share experiences and offer mutual support [36]. Moreover, psychosocial, leisure courses, awareness-raising initiatives to foster empathy in society, and reintegration programs to facilitate returning to work may be beneficial [36–39]. Future research should investigate whether these findings also apply to other countries.

## Supplementary Information

Below is the link to the electronic supplementary material.

Supplementary Material 1

## Data Availability

The data used in this study are third-party data. The anonymized data sets of the DEAS (1996, 2002, 2008, 2011, 2014, 2017, 2020, 2020/2021, 2023) are available for secondary analysis. The data has been made available to scientists at universities and research institutes exclusively for scientific purposes. The use of data is subject to written data protection agreements. Microdata of the German Ageing Survey (DEAS) are available free of charge to scientific researchers for non-profitable purposes. The FDZ-DZA provides access and support to scholars interested in using DEAS for their research. However, for reasons of data protection, signing a data distribution contract is required before data can be obtained. For further information on the data distribution contract, please see https://www.dza.de/en/research/fdz/access-to-data/application (accessed on 10 June 2025).
