# Supplementary material for: Long COVID and psychosocial factors among middle-aged and older adults. Results of the nationally representative German Ageing Survey
Source: Aging Clin Exp Res. 2025 Nov 5;37(1):313. doi: 10.1007/s40520-025-03246-7 (PMC12589248; doi:10.1007/s40520-025-03246-7)
Supplement: Supplementary file 1 — Supplementary Material 1 [file 40520_2025_3246_MOESM1_ESM.docx]

**Supplementary Table 1.** Association of long COVID with psychosocial outcomes stratified by sex. Results of linear regressions (DEAS, wave 8, weighted)

|  |  |  |  |  |  |  |  |  |
| --- | --- | --- | --- | --- | --- | --- | --- | --- |
|  | Depressive symptoms among men | Loneliness among men | Perceived social isolation among men | Life satisfaction among men | Depressive symptoms among women | Loneliness among women | Perceived social isolation among women | Life satisfaction among women |
|  |  |  |  |  |  |  |  |  |
|  |  |  |  |  |  |  |  |  |
| Long COVID: Presence (Reference category: Absence) | 1.67 | -0.03 | 0.09 | -0.08 | 1.79 | 0.18 | 0.37*** | -0.07 |
|  | (-0.32 - 3.66) | (-0.24 - 0.18) | (-0.21 - 0.38) | (-0.43 - 0.26) | (-1.84 - 5.41) | (-0.06 - 0.41) | (0.20 - 0.55) | (-0.38 - 0.23) |
|  |  |  |  |  |  |  |  |  |
|  |  |  |  |  |  |  |  |  |
| Sociodemographic covariates | 🗸 | 🗸 | 🗸 | 🗸 | 🗸 | 🗸 | 🗸 | 🗸 |
|  |  |  |  |  |  |  |  |  |
| Lifestyle-related covariates | 🗸 | 🗸 | 🗸 | 🗸 | 🗸 | 🗸 | 🗸 | 🗸 |
|  |  |  |  |  |  |  |  |  |
| Health-related covariates | 🗸 | 🗸 | 🗸 | 🗸 | 🗸 | 🗸 | 🗸 | 🗸 |
|  |  |  |  |  |  |  |  |  |
| Individuals | 1,922 | 1,912 | 1,912 | 1,913 | 2,095 | 2,077 | 2,081 | 2,086 |
| R² | 0.24 | 0.13 | 0.16 | 0.38 | 0.35 | 0.17 | 0.21 | 0.30 |

Comments: Unstandardized beta coefficients are shown, with 95% CI in parentheses; *** p<0.001, ** p<0.01, * p<0.05, + p<0.10.

Sociodemographic covariates include: age, sex, marital status, labor force participation, and education; lifestyle-related covariates include: alcohol intake, sports activity, and smoking behavior; health-related covariates include: self-rated health, physical functioning, and the number of chronic conditions.
